# The impact of parenting styles on physical activity among adolescents: the mediating role of psychological resilience

**DOI:** 10.7717/peerj.20981

**Published:** 2026-03-17

**Authors:** Jiang Zhu, Donglin Hu

**Affiliations:** 1 College of Physical Education, Jiangsu Ocean University, Lianyungang, Jiangsu Province, China; 2 College of Sports Industry and Leisure, Nanjing Sport Institute, Nanjing, Jiangsu Province, China; 3 The Department of Physical Education, Nanjing Agricultural University, Nanjing, Jiangsu Province, China; 4 School of Physical Education and Educational Science, Tianjin University of Sport, Tianjin, China

**Keywords:** Parenting styles, Psychological resilience, Adolescents, Mediation, PA

## Abstract

**Objective:**

To evaluate the impact of parenting styles and psychological resilience on physical activity (PA) levels in junior high school students, and to examine the mediating role of resilience in this relationship.

**Methods:**

A cross-sectional survey was conducted among 336 adolescents (195 boys, 141 girls) from Nanjing, Yangzhou, and Lianyungang in Jiangsu Province, China. Data were collected using the Chinese versions of the Physical Activity Questionnaire for Adolescents (PAQ-A), the Short-Form Egna Minnen Beträffande Uppfostran for Children (s-EMBU-C), and the Adolescent Resilience Scale. A one-way analysis of variance (ANOVA), Pearson correlation coefficients, logistic regression, and hierarchical multiple regression were used to explore the associations among variables. Mediation analyses were conducted using the PROCESS macro (Model 4, 5,000 bootstraps samples).

**Results:**

(1) Gender and grade differences in PA were significant: girls were more likely to be in the low-PA group, while boys were more likely to be in the medium- or high PA groups. As grade level increased, the proportion of students in the low-PA group decreased, the medium-PA group increased, and the high PA remained stable (*p* < 0.01). No urban-rural differences were observed. (2) Parenting styles were significantly associated with PA levels: parental emotional warmth was positively correlated with PA, whereas paternal rejection was negatively correlated; overprotection showed weaker but still significant effects. (3) Hierarchical regression revealed that parental emotional warmth was a strong positive predictor of PA, while paternal rejection was a negative predictor. The final model explained 49.8% of the variance in total PAQ scores. (4) Psychological resilience mediated the relationship between parenting styles and PA. Emotional warmth had both direct and indirect effects on PA through resilience, while paternal rejection and overprotection influenced PA indirectly, with paternal rejection exerting the strongest negative indirect effect.

**Conclusion:**

Greater parental emotional warmth and lower levels of paternal rejection are associated with increased PA levels among adolescents, with psychological resilience acting as a key mediator. These findings underscore the importance of strategies that promote positive parenting and resilience to enhance PA and support adolescent development.

## Introduction

Physical activity (PA) during adolescence is critical for physical health, psychological well-being, and social development (Xu et al., 2025). The World Health Organization (WHO) recommends that children and adolescents aged 5–17 engage in at least 60 min of moderate-to-vigorous aerobic activity daily, along with muscle- and bone-strengthening activities three times per week (WHO, 2020). Adhering to these guidelines significantly reduces the risks of obesity, metabolic diseases, and psychological disorders, while enhancing mental well-being and academic engagement (WHO, 2020). However, increasing academic pressure, digital lifestyles, and limited access to exercise resources have led to a global failure to meet these recommended guidelines (WHO, 2019). Recent global surveillance studies have similarly reported that more than 80% of adolescents worldwide fail to achieve the recommended PA levels, highlighting a pervasive decline in youth activity across diverse cultural and socioeconomic contexts (Guthold et al., 2020; Sallis et al., 2016). In China, a pooled analysis of national surveillance data (2017–2019) found that only 28.7% of children and adolescents achieved the WHO-recommended ≥60 min of daily moderate-to-vigorous physical activity (MVPA), with girls, older students, and those from rural areas showing even lower compliance, indicating significant gender and urban-rural disparities (Guo, Zhu & Wang, 2024; Xu & Gao, 2018). Despite efforts to promote school-based physical education and extracurricular PA, improving adolescent PA at the family and individual levels remains an urgent challenge (Lin & Wang, 2025).

Among the various ecological factors influencing adolescent PA, parenting styles and psychological resilience are particularly crucial (Qiu et al., 2025; Wang et al., 2024). Systematic reviews and longitudinal studies have highlighted that parental emotional support, role modeling, and active involvement in sports significantly enhance adolescents’ motivation and persistence in PA by offering opportunities, establishing behavioral norms, and fostering emotional security (Çakmak Tolan & Bolluk Uğur, 2024; Qiu et al., 2025). International meta-analyses have similarly demonstrated robust associations between supportive parenting practices and youth PA across Western and non-Western cultural contexts, indicating that parental behaviors play a universally important role in shaping adolescents’ activity patterns (Biddle et al., 2019; Rhodes, Perdew & Malli, 2020). Resilience, defined as the psychological capacity to maintain or regain adaptive functioning under stress, has been linked to higher PA levels and can be enhanced through exercise interventions (Husain et al., 2024; Zou & Liu, 2025). Previous studies suggest that PA interventions not only enhance resilience in adolescents but also imply that that resilience may serve as a mediator in the relationship between parenting styles and adolescents’ PA behaviors (Guo & Liang, 2023; Peng et al., 2025; Zhao & Lipowski, 2025). Similar findings have been reported in international research, where resilience-related psychological resources are strongly linked to adolescents’ participation in physical activities and their adaptive functioning (Belcher et al., 2021; Fergus & Zimmerman, 2005). However, evidence on the mechanisms connecting family and psychological factors to adolescent PA is limited in China. Although these mechanisms have been extensively examined in Western populations, there is less understanding of whether similar pathways exist among Chinese adolescents, particularly considering cultural differences in parenting norms and family socialization. Junior high school students, a critical developmental group, face an accelerated academic pace, a stronger peer culture, and rapid yet heterogeneous physical and psychological growth. Their PA behaviors are particularly susceptible to family emotional climate-specifically, perceived parental warmth and rejection-as well as parental control or overprotection (Zhang et al., 2024b). Additionally, PA participation may be influenced by specific resilience dimensions, including positive cognition, goal focus, and emotional regulation (Dong et al., 2023; Liu, 2025). Consequently, examining the interactive mechanisms of parenting styles and resilience in shaping PA in junior high school students holds both theoretical and practical significance.

The present study recruited junior high school students from Nanjing, Yangzhou, and Lianyungang in Jiangsu Province. PA was assessed using the Chinese version of the Physical Activity Questionnaire for Adolescents (PAQ-A) (Li, Wang & Li, 2015). Parenting styles were measured using the Short-Form Egna Minnen Beträffande Uppfostran for Children (s-EMBU-C, Chinese version), which assesses three dimensions: emotional warmth, overprotection, and rejection (Jiang et al., 2010). Psychological resilience was evaluated using the Chinese version of the Adolescent Resilience Scale (Hu & Gan, 2008). The study aimed to (1) compare differences in parenting styles and resilience across PA groups, (2) examine the correlation patterns among parenting styles, resilience, and PA, and (3) test whether resilience mediates the relationship between parenting styles and PA, while accounting for demographic factors such as gender and residential background.

Accordingly, we proposed the following hypotheses:

H1: Parental emotional warmth is positively associated with adolescents’ PA.

H2: Parental rejection is negatively associated with adolescents’ PA.

H3: The relationship between parental overprotection and adolescents’ PA is complex, potentially weak or inconsistent.

H4: Resilience mediates the relationship between parenting styles and adolescents’ PA.

H5: Gender and residential background are associated with PA differences, with girls and rural students more likely to have lower PA levels.

In summary, this study aims to enhance the theoretical understanding of how family socialization and individual psychological resources interact to shape adolescent PA behaviors, while providing practical implications for family education, school-based physical education programs, and resilience-promoting interventions to improve PA levels and health in junior high school students.

## Methods and Participants

### Study design

This study employed a cross-sectional survey design to examine the relationships between PA, parenting styles, and psychological resilience among junior high school students. Data were collected using standardized questionnaires.

#### Questionnaires

##### Physical Activity Questionnaire for Adolescents (PAQ-A, Chinese version).

This instrument assesses the frequency and intensity of MVPA over the past seven days. Scores range from 1 to 5, with higher scores indicating higher PA levels. Participants were categorized into three PA levels based on tertile cutoffs of the sample distribution: low (<2.71), medium (2.71−3.29), and high (>3.29). Tertile-based classification was used to group adolescents into low, medium, and high PA levels based on the sample distribution, which is a common used analytic strategy when validated clinical cutoffs for questionnaire scores are unavailable. The PAQ-A demonstrated acceptable internal consistency in the present sample (Cronbach’s *α* = 0.76).

##### Short-Form Egna Minnen Beträffande Uppfostran for children (s-EMBU-C, Chinese version).

This scale evaluates three dimensions of parenting styles-emotional warmth, overprotection, and rejection-with separate versions for mothers and fathers. It has demonstrated good reliability and validity among Chinese adolescents. In this study, the emotional warmth, overprotection, and rejection subscales showed good reliability, with Cronbach’s *α* values ranging from 0.77 to 0.85 across both mother and father versions.

##### Adolescent resilience scale (Chinese version).

This instrument assesses five dimensions of resilience: positive cognition, emotional control, family support, goal focus, and interpersonal assistance. Higher scores reflect higher resilience levels. The five resilience subscales demonstrated good internal consistency in the present sample, with Cronbach’s *α* values ranging from 0.79 to 0.86.

#### Data analysis

All analyses were performed using SPSS version 26.0.

Descriptive statistics and reliability tests were conducted, calculating means, standard deviations, and Cronbach’s *α* to assess internal consistency.

A one-way analysis of variance (ANOVA) was used to compare parenting styles and resilience across PA levels, with *η*^2^ as the effect size.

Pearson correlation analysis was used to examine the associations between parenting styles, resilience, and PA. Correlation analyses were exploratory in nature, and no adjustment for multiple comparisons was applied.

Multiple regression analysis was performed to test the predictive effects of parenting styles (emotional warmth, overprotection, rejection) and resilience on PA, controlling for gender and residential background. Multicollinearity was assessed using variance inflation factors (VIF), all of which were below 2, indicating no serious multicollinearity among predictors.

Mediation analysis was performed using the PROCESS macro (Model 4, 5,000 bootstrap samples) to assess whether resilience mediated the relationship between parenting styles and PA.

Although cross-sectional mediation cannot establish causality, the PROCESS macro was used to estimate statistical indirect associations, which is an accepted analytic approach in cross-sectional behavioral research. Bootstrapping with 5,000 samples was employed to estimate indirect effects because bootstrapping does not require the indirect effect to follow a normal distribution (Hayes, 2013; Preacher & Hayes, 2004). Model diagnostics indicated no serious violations of linear regression assumptions, including normality, linearity, and the absence of influential cases.

Questionnaires with more than 10% missing items were excluded, while those with fewer missing items (<10%) were handled using within-subscale mean substitution, a commonly used approach for Likert-type data. Outliers were identified using a ±3 standard deviation (SD) criterion, and no extreme outliers were detected. To address potential limitations of mean-based outlier detection, we further conducted regression influence diagnostics, including inspection of standardized residuals, leverage values, and Cook’s distance. No influential cases were identified, indicating the absence of problematic multivariate outliers.

All tests were two-tailed, with significance set at *p* < 0.05. A *post-hoc* power analysis (*α* = .05, f^2^ = .15) was conducted to evaluate whether the sample size was adequate for detecting medium effects. The final sample of 336 participants provided statistical power greater than 0.90 for the multiple regression models used in this study, suggesting that the sample size was adequate for detecting medium effects.

### Participants

#### Study sites

The study was conducted in junior high schools across Nanjing, Yangzhou, and Lianyungang, Jiangsu Province, representing distinct socioeconomic and educational contexts: Nanjing (highly developed, southern Jiangsu), Yangzhou (moderately developed, central Jiangsu), and Lianyungang (relatively underdeveloped, northern Jiangsu). This cross-regional design provided a comprehensive understanding of PA behaviors among adolescents in diverse contexts.

#### Sampling

##### Sampling method.

Stratified random sampling combined with convenience sampling was employed. Schools were randomly selected from each city, with two grades chosen per school. Stratified random sampling was applied at the school and grade levels, while class selection was based on feasibility and convenience due to fixed teaching schedules and administrative constraints-a common and accepted practice in school-based research. Inclusion criteria: students to be enrolled in Grades 7–9 and capable of completing the questionnaire independently; students with cognitive or physical conditions that prevented them from completing the questionnaire were excluded.

##### Sample size.

A total of 336 valid responses were collected, comprising 195 boys and 141 girls, with 200 students from urban areas and 136 from rural areas. Participants ranged in age from 12 to 15 years, corresponding to the typical junior high school age group.

##### Demographic balance.

The gender ratio was balanced, and both urban and rural students were represented, reflecting the demographic characteristics of adolescents in the study regions.

##### Inclusion criteria.

students were required students to be enrolled in Grades 7–9 and capable of completing the questionnaire independently; students with cognitive or physical conditions that prevented them from completing the questionnaire were excluded.

#### Data collection

Data were collected from September to December 2024. Paper-based questionnaires were distributed and collected during class, while online surveys were administered through school platforms to ensure data completeness and representativeness.

To minimize potential mode-related bias, both paper-based and online questionnaires followed identical wording, instructions, administration procedures, and time limits. All surveys were completed under teacher supervision to ensure standardized testing conditions.

A total of 360 questionnaires were distributed, with 336 valid responses obtained after excluding incomplete surveys, resulting in a response rate of 93.3%.

### Ethical considerations

All participants and their guardians provided written informed consent, which included clear information regarding voluntary participation and the right to withdraw. Data were anonymized using coded identifiers to ensure confidentiality and were used exclusively for research purposes. The study adhered to the ethical principles outlined in the Declaration of Helsinki and was approved by the Ethics Committee of Tianjin University of Sport (Approval No. 2025100). The study also complied with national guidelines for research involving minors in China.

## Results

### Distribution of PA levels across demographic characteristics

**Figure 1:**
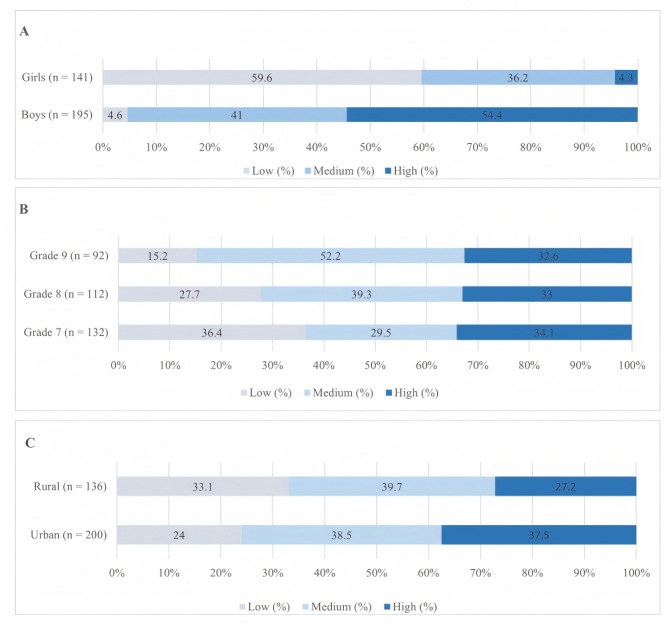
Distribution of physical activity levels across demographic variables. Note: (A) Distribution of PA levels by gender; (B) distribution of PA levels across grade levels; (C) distribution of PA levels by residential background. PA levels were classified into low, medium, and high groups based on tertiles of the PAQ-A total score. Values are presented as percentages.

Participants (*n* = 336) were classified into three PA groups based on tertile cutoffs of the PAQ-A total score (range 1–5): low PA (<2.71; *n* = 93; 27.7%), medium PA (2.71–3.29; *n* = 131; 39.0%), and high PA (>3.29; *n* = 112; 33.3%). Figure 1 illustrates the distribution of PA levels across gender, grade, and residential background. Chi-square tests revealed significant differences in PA levels by gender and grade, but no significant differences by residential background (Fig. 1).

Boys were predominantly in the medium (41.0%) and high PA groups (54.4%), while girls were mainly in the low PA group (59.6%) and few in the high PA group (4.3%) (*χ*^2^ = 151.42, *df* = 2, *p* < 0.001, Cramér’s *V* = 0.67), indicating a large effect size.

The proportion of low PA decreased with grade level (36.4% in Grade 7, 27.7% in Grade 8, and 15.2% in Grade 9), while medium PA increased (29.5%, 39.3%, and 52.2%, respectively). The proportion of high PA remained stable at ∼33% across grades (*χ*^2^ = 15.92, *df* = 4, *p* = 0.003, Cramér’s *V* = 0.15), suggesting a small-to-moderate association.

No significant differences in PA levels by residential background were found (*χ*^2^ = 5.02, *df* = 2, *p* = 0.081, Cramér’s *V* = 0.12), with urban students showing 24.0%, 38.5%, and 37.5% in the low, medium, and high PA groups, respectively, and rural students showing 33.1%, 39.7%, and 27.2%, reflecting a small effect size.

### Differences in parenting styles and resilience across **PAlevels**

One-way ANOVA results are presented in Table 1.

**Table 1:** Parenting styles and psychological resilience across physical activity levels (*N* = 336).

**PA level**	**n**	**Maternal emotional warmth**	**Maternal overprotection**	**Maternal rejection**	**Paternal emotional warmth**	**Paternal overprotection**	**Paternal rejection**	**Positive cognition**	**Emotional control**	**Family support**	**Goal focus**	**Interpersonal assistance**
Low	93	16.34 ± 3.85^a^	13.22 ± 5.24^a^	11.09 ± 5.76^a^	15.10 ± 4.50^a^	13.10 ± 4.97^a^	14.39 ± 4.14^a^	8.98 ± 3.55^a^	13.29 ± 5.32^a^	14.09 ± 4.91^a^	11.29 ± 4.24^a^	13.38 ± 5.19^a^
Medium	131	21.00 ± 1.75^b^	15.33 ± 4.90^b^	9.50 ± 2.27^b^	21.03 ± 1.63^b^	14.53 ± 3.92^b^	10.47 ± 2.48^b^	13.49 ± 1.15^b^	20.54 ± 1.72^b^	20.53 ± 1.76^b^	16.79 ± 1.41^b^	20.67 ± 1.69^b^
High	112	21.73 ± 2.37^c^	15.46 ± 3.98^b^	9.18 ± 2.66^b^	21.63 ± 2.70^b^	15.54 ± 4.72^b^	10.52 ± 2.71^b^	14.01 ± 1.88^c^	21.61 ± 3.00^c^	21.19 ± 2.66^b^	17.69 ± 2.34^c^	21.65 ± 2.67^c^
F		119.34	7.19	7.72	144.16	7.54	53.24	147.99	171.27	150.99	158.90	186.99
p		<0.001	0.001	0.001	<0.001	0.001	<0.001	<0.001	<0.001	<0.001	<0.001	<0.001
*η* ^2^		0.417	0.041	0.044	0.464	0.043	0.242	0.472	0.507	0.476	0.488	0.529

**Notes.**

Different superscript letters (^abc^) within the same column indicate significant differences between groups (*p* < 0.05, LSD *post-hoc* test). *η*^2^ = effect size.

Maternal emotional warmth varied significantly across PA groups (*F* = 119.34, *p* < 0.001, *η*^2^ = 0.417), with higher scores associated with higher PA levels. Maternal rejection also differed significantly (*F* = 7.72, *p* = 0.001, *η*^2^ = 0.044), with the low PA group scoring highest and the high PA group scoring lowest. Maternal overprotection showed significant differences as well (*F* = 7.19, *p* = 0.001, *η*^2^ = 0.041), with the low PA group scoring lower than the medium and high PA groups.

For paternal parenting, emotional warmth differed significantly across groups (*F* = 144.16, *p* < 0.001, *η*^2^ = 0.464), with the high PA group scoring highest. Paternal rejection also showed significant differences (*F* = 53.24, *p* < 0.001, *η*^2^ = 0.242), with the low PA group scoring highest and the high PA group lowest. Paternal overprotection was significantly different (*F* = 7.54, *p* = 0.001, *η*^2^ = 0.043), with the low PA group scoring lowest and the medium/high PA groups scoring higher.

Psychological resilience dimensions-positive cognition, emotional control, family support, goal focus, and interpersonal assistance-all differed significantly across PA groups (Fs = 147.99–186.99, *p* < 0.001). Effect sizes were large (*η*^2^ = 0.472–0.529), with resilience increasing steadily with PA levels.

### Correlation analysis

Pearson correlation analysis showed positive associations between the total PAQ score and parental emotional warmth, as well as all dimensions of psychological resilience (*r* = .61–.71). The PAQ score was negatively associated with parental rejection, with stronger associations observed for paternal rejection (*r* = −.41) than for maternal rejection (*r* = −.21). Small to moderate positive associations were also observed between the PAQ score and parental overprotection (maternal *r* = .12; paternal *r* = .21).

All correlation coefficients are presented for descriptive purposes only and should be interpreted with caution (Table 2).

**Table 2:** Pearson correlations among study variables (*N* = 336).

**Variable**	**1**	**2**	**3**	**4**	**5**	**6**	**7**	**8**	**9**	**10**	**11**	**12**
1. PAQ total score	1.00											
2. Maternal emotional warmth	.61	1.00										
3. Maternal rejection	−.21	−.42	1.00									
4. Maternal overprotection	.12	.16	−.10	1.00								
5. Paternal emotional warmth	.65	.62	−.18	.18	1.00							
6. Paternal overprotection	.21	.20	.02	.52	.19	1.00						
7. Paternal rejection	−.41	−.45	.15	−.14	−.44	−.14	1.00					
8. Positive cognition	.68	.70	−.24	.16	.72	.18	−.49	1.00				
9. Emotional control	.71	.67	−.20	.18	.73	.18	−.53	.79	1.00			
10. Family support	.65	.70	−.20	.15	.74	.17	−.53	.78	.80	1.00		
11. Goal focus	.68	.70	−.27	.20	.71	.18	−.53	.77	.78	.80	1.00	
12. Interpersonal assistance	.70	.67	−.21	.20	.70	.21	−.52	.78	.79	.80	.78	1.00

**Notes.**

Values are Pearson correlation coefficients. All correlations are reported for descriptive purposes only and should be interpreted with caution. No correction for multiple testing was applied.

### Predicting PA levels by gender and residence

Binary logistic regression revealed a significant overall model (*χ*^2^ = 135.28, *df* = 2, *p* < 0.001) with a Nagelkerke R^2^ of 0.478, indicating good explanatory power. Gender was a significant predictor, with females significantly less likely than males to fall into the moderate/high PA group (B = −3.42, *p* < 0.001, Exp(B) = 0.03). Place of residence (urban *vs.* rural) was not significant after controlling for gender (B = −0.45, *p* = 0.151, Exp(B) = 0.64) (Table 3). The model showed acceptable fit, as indicated by a non-significant Hosmer–Lemeshow test (*p* = 0.449) and an overall classification accuracy of 81.0%.

**Table 3:** Binary logistic regression predicting moderate/high physical activity (*N* = 336).

**Variable**	**B**	**SE**	**Wald**	**df**	***p***	**Exp(B)**
Gender	−3.42	0.38	79.44	1	<0.001	0.03
Residence	−0.45	0.31	2.07	1	0.151	0.64
Constant	6.64	0.73	83.89	1	<0.001	762.56

**Notes.**

Dependent variable = physical activity level (0 = low, 1 = moderate/high). Gender was coded as 0 = male, 1 = female. Residence was coded as 0 = urban, 1 = rural. Exp(B) represents the odds ratio.

### Predicting PA by parenting styles

Hierarchical multiple regression analysis yielded the following results:

In Model 1, father’s emotional warmth was a significant positive predictor of the PAQ total score (β = 0.649, *p* < 0.001), explaining 42.1% of the variance.

In Model 2, the inclusion of mother’s emotional warmth increased the explained variance to 49.2% (Δ*R*^2^ = 0.071, *p* < 0.001), with both father’s and mother’s emotional warmth significantly predicting PAQ scores.

In Model 3, incorporating father’s rejection resulted in a small but significant improvement in explanatory power (Δ*R*^2^ = 0.006, *p* = 0.049), with father’s rejection negatively predicting PAQ scores (β = −0.089, *p* = 0.049). Both parents’ emotional warmth remained significant positive predictors. Although the increase in explained variance was statistically significant, the magnitude of this increment (Δ*R*^2^ = 0.006) was small and should therefore be interpreted with caution, suggesting that father’s rejection contributes modest additional explanatory value beyond parental emotional warmth.

The final model explained 49.8% of the variance in PAQ scores, highlighting the strong positive role of parental emotional warmth in adolescents’ PA and the negative influence of father’s rejection (Table 4).

**Table 4:** Hierarchical regression of parenting styles predicting physical activity (*N* = 336).

**Model**	**Variable**	**B**	**SE**	β	**t**	*p*	**95% CI**	**R^2^**	ΔR^2^	**F**
1	Constant	1.158	0.125	–	9.285	<0.001	0.913–1.403	0.421	–	242.665
	Father Emotional Warmth	0.097	0.006	0.649	15.578	<0.001	0.085–0.109			
2	Constant	0.587	0.144	–	4.078	<0.001	0.304–0.870	0.492	0.071^***^	161.008
	Father Emotional Warmth	0.065	0.007	0.438	8.772	<0.001	0.051–0.080			
	Mother Emotional Warmth	0.060	0.009	0.340	6.810	<0.001	0.042–0.077			
3	Constant	0.923	0.222	–	4.155	<0.001	0.486–1.361	0.498	0.006^*^	109.587
	Father Emotional Warmth	0.062	0.008	0.415	8.129	<0.001	0.047–0.077			
	Mother Emotional Warmth	0.055	0.009	0.314	6.106	<0.001	0.037–0.073			
	Father Rejection	−0.015	0.008	−0.089	−1.980	0.049	−0.031–0.000			

**Notes.**

* Dependent variable, PAQ total score; B, unstandardized coefficients; SE, standard error; β, standardized coefficients; CI, confidence interval; R^2^, cumulative explained variance; ΔR^2^, change in R^2^.

*** *p* < 0.001; *p* < 0.05.

To ensure the robustness of the regression results, we conducted multicollinearity diagnostics. All variance inflation factor (VIF) values for the predictors were below 2.0, indicating no concerns regarding multicollinearity.

### Mediating role of resilience

**Figure 2:**
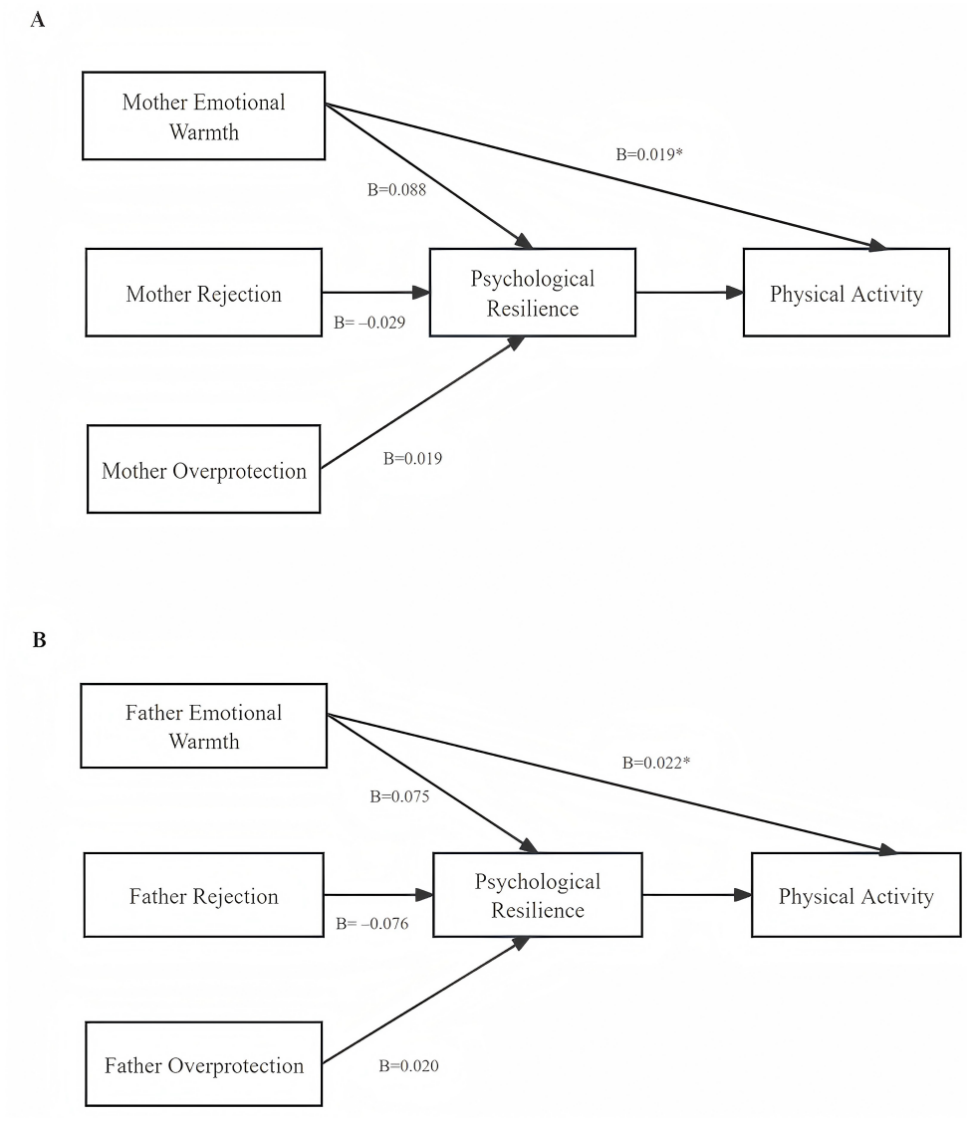
Mediation models of psychological resilience between parenting styles and physical activity. Note: (A) Maternal parenting styles; (B) Paternal parenting styles. Emotional warmth, rejection, and overprotection were included as parenting-style indicators. Values represent unstandardized coefficients (B). Indirect effects were estimated using 5,000 bootstrap samples. Mediation effects were considered statistically supported when the 95% confidence interval did not include zero. ^∗^*p* < .05.

Mediation analysis indicated that parental emotional warmth was statistically associated with both direct and indirect paths to PA through resilience, consistent with a pattern of partial mediation. In contrast, the direct effects of parental rejection and overprotection were not significant, but both influenced PA indirectly *via* resilience, indicating full mediation. Notably, the negative indirect effect of father’s rejection was the strongest (B = −0.076, 95% CI [−0.088 to −0.063]), with father’s rejection primarily reducing PA by weakening resilience. Interestingly, parental overprotection showed a small positive indirect association with PA through resilience, despite non-significant direct paths (Fig. 2).

## Discussion

Using a multi-site school-based sample, this study explored the relationships between parenting styles, psychological resilience, and adolescents’ PA levels among junior high school students in Jiangsu Province. Overall, PA differed by gender and grade, and parenting styles and resilience-related psychological resources showed consistent associations with PA. Specifically, (1) gender and grade level were significant factors influencing PA; (2) parental warmth, rejection, and resilience dimensions varied significantly across PA groups; (3) parental warmth was positively associated with higher PA, while paternal rejection was negatively associated with PA; and (4) resilience may represent a statistical pathway linking parenting styles and PA.

### Gender and grade differences

The results showed that girls were more likely to engage in low-level PA, while boys were more likely to be represented in the moderate and high-level PA groups. This pattern aligns with previous research indicating that adolescent girls are generally less active than boys, likely due to body image concerns, academic pressure, and lower exercise self-efficacy (Dumith et al., 2011; Rosselli et al., 2020). Grade-level differences revealed that the proportion of low PA decreased and moderate PA increased as grade level increased, while high PA remained stable. These trends are consistent with longitudinal findings showing that PA tends to decline with age during adolescence, while sedentary behaviors increase (Caspersen, Pereira & Curran, 2000; Farooq et al., 2020). Taken together, these findings highlight the importance of sustained school-based PA opportunities, with particular attention to girls and students in higher grades.

### The role of parenting styles

Parental warmth was strongly positively associated with adolescents’ PA, explaining a significant proportion of variance in the regression models. This finding supports previous evidence that parental warmth and support are linked to adolescents’ well-being and engagement in healthy behaviors, including PA (Bülow et al., 2021; Edwardson & Gorely, 2010). In contrast, paternal rejection showed a negative association with PA, consistent with research indicating that negative parenting behaviors undermine adolescents’ self-efficacy and persistence in PA (Sleddens et al., 2011). While parental overprotection also differed across PA groups, the relatively small effect size suggests a more complex and context-dependent mechanism rather than a straightforward association. These results also have cross-cultural relevance. Parenting behaviors such as control or overprotection may be interpreted differently across cultures: in many Western settings they are more likely to be viewed as autonomy-thwarting, whereas in Chinese contexts they may be perceived as normative involvement or protection. Such cultural differences in meaning may partly explain the relatively weak or mixed associations observed for overprotection, compared with the more consistent effects of warmth and rejection.

### The role and mediating effect of resilience

This study revealed that adolescents with higher PA levels demonstrated more favorable psychological profiles, including stronger positive cognition, emotional control, family support, goal orientation, and interpersonal assistance. This pattern aligns with prior research showing that PA interventions can enhance these psychological resources, which in turn support sustained PA engagement (Qiu et al., 2025; Zhao & Lipowski, 2025). Mediation analysis further indicated that these psychological attributes may represent an important statistical pathway linking parenting styles to PA. Specifically, parental warmth showed a direct positive association with PA and was also indirectly associated with higher PA through its associations with adolescents’ adaptive psychological capacities. In contrast, parental rejection and overprotection had no significant direct effects but were indirectly associated with PA through their relationships with resilience-related psychological resources. Notably, paternal rejection exhibited the strongest adverse indirect link, suggesting a particularly detrimental role. These findings are consistent with socio-ecological models emphasizing the interplay of family context, psychological processes, and health behaviors (Bronfenbrenner, 1994; Fergus & Zimmerman, 2005). However, given the cross-sectional design, these pathways should be interpreted as statistical associations rather than causal mechanisms.

### Theoretical and practical implications

The findings of this study have both theoretical and practical implications. Theoretically, they contribute to the growing body of evidence on how family socialization and psychological resources collectively influence adolescent PA (Wang et al., 2024; Zhang et al., 2024a). By simultaneously examining parenting styles, resilience, and PA within a regionally diverse Chinese adolescent sample, this study provides cross-cultural evidence that extends existing models predominantly derived from Western contexts.

Practically, the distinct effects of parental warmth and rejection underscore the importance of family-based education in promoting supportive parenting practices. Additionally, the mediating role of resilience suggests that interventions combining resilience training (*e.g.*, goal-setting, emotion regulation, interpersonal cooperation) with PA promotion could offer dual benefits. Such integrated approaches are increasingly recommended in health promotion literature for improving both mental health and PA outcomes (Lubans et al., 2016). The observed gender and grade differences further highlight the need for targeted and developmentally sensitive school-based PA programs, particularly for girls and older students.

### Summary of hypothesis testing

The study proposed five hypotheses in the introduction. Results supported for H1 (positive link between parental warmth and PA), H2 (negative link between parental rejection and PA), and H4 (mediating role of resilience). H3 (complex role of overprotection) was partially supported, as overprotection had no significant direct effect but exerted indirect effects through resilience. H5 (differences by gender and residence) was partially supported, with significant gender differences but no significant urban–rural differences. Overall, the findings were broadly consistent with the proposed conceptual framework, highlighting the interconnected roles of parenting styles, resilience, and adolescent PA.

### Limitations and future directions

Several limitations should be acknowledged. First, the cross-sectional design limits causal inference, and longitudinal or experimental studies are necessary to validate the observed pathways. To strengthen conceptual clarity, the present findings should therefore be interpreted as associations rather than causal effects, and future research should aim to clarify the temporal ordering among parenting styles, resilience, and PA by adopting designs that allow for the examination of change over time.

Second, PA was assessed through self-reported questionnaires, which may introduce bias; future studies should incorporate objective measures, such as accelerometers.

Third, the sample was restricted to three cities in Jiangsu Province, limiting the generalizability; although the multi-site design increases contextual diversity, broader national samples are still needed. In addition, potential confounders not captured in the present study—such as socioeconomic status, parental education, peer influences, and school environment—should be considered in future models to enhance explanatory precision.

Furthermore, the relatively large effect sizes observed in certain analyses (*e.g.*, *η*^2^ for resilience dimensions) may partly reflect unmeasured covariates, underscoring the need for future models to include socioeconomic status, peer influences, and school-level factors to reduce potential inflation of effect estimates.

Intervention-based studies targeting both family involvement and resilience-enhancing strategies may further clarify how these factors jointly relate to sustained improvements in adolescent PA.

## Conclusion

This study found that gender and grade level significantly influenced adolescents’ PA. Girls were more likely to belong to the low PA group, while boys were more frequently in the moderate and high PA groups. As grade level increased, the proportion of students in the low PA group decreased, while those in the moderate PA group increased.

In contrast, no significant urban–rural differences were observed, and thus, we refrain from making interpretations beyond what the data support.

Parenting styles were strongly associated with PA: parental warmth was positively associated with PA participation, whereas paternal rejection was negatively associated with it, and the role of overprotection appeared more complex. Resilience was identified as a mediating correlate, linking parenting styles and PA, reflecting adolescents’ psychological resources and how family factors influence behavioral patterns.

These findings should be interpreted as statistical associations rather than causal relationships owing to the cross-sectional design.

Taken together, the results suggest that cultivating a supportive family environment, reducing negative parenting practices, and strengthening adolescents’ resilience may be associated with higher PA levels, although longitudinal and intervention-based studies are needed to confirm the directionality of these relationships.

## Supplemental Information

10.7717/peerj.20981/supp-1 Supplemental Information 1 Summary of measurement data from all scales

10.7717/peerj.20981/supp-2 Supplemental Information 2 Data coding and categorization for variables

## Additional Information and Declarations

### Data Availability

The following information was supplied regarding data availability: The data is available in the Supplemental File and at Zenodo: Zhu, J., & Donglin, H. (2025). Dataset for ”Parenting Styles, Resilience, and Physical Activity among Chinese Adolescents” [Data set]. Zenodo. https://doi.org/10.5281/zenodo.17694162.
