# Supplementary material for: The impact of parenting styles on physical activity among adolescents: the mediating role of psychological resilience
Source: PeerJ. 2026 Mar 17;14:e20981. doi: 10.7717/peerj.20981 (PMC13003944; doi:10.7717/peerj.20981)
Supplement: Supplemental Information 2 [file peerj-14-20981-s002.docx]

**Data Coding Description (Codebook)**

**1. Gender**

1 = Male
2 = Female

**2. Grade**

1 = 1st Year of Junior High
2 = 2nd Year of Junior High
3 = 3rd Year of Junior High

**3. PAQ_Level**

1 = Low Activity Level
2 = Moderate Activity Level
3 = High Activity Level

**4. Originnumber**

0 = Urban
1 = Rural

**5. PAQ_LEVEL**

1 = Low
2 = Medium
3 = High
